# Child dental neglect and legal protections: a compendium of briefs from policy reviews in 26 countries and a special administrative region of China

**DOI:** 10.3389/froh.2023.1211242

**Published:** 2023-10-20

**Authors:** Moréniké Oluwátóyìn Foláyan, Francisco Ramos-Gomez, Olawunmi Adedoyin Fatusi, Nouran Nabil, Germana V. Lyimo, Irene Kida Minja, Ray M. Masumo, Nadia Mohamed, Nicoline Potgieter, Cleopatra Matanhire, Pamela Maposa, Chiedza Runyararo Akino, Abiola Adeniyi, Simin Z. Mohebbi, Passent Ellakany, Jieyi Chen, Rosa Amalia, Alfredo Iandolo, Faizal C. Peedikayil, Athira Aravind, Ola B. Al-Batayneh, Yousef S. Khader, Sadeq Ali Al-Maweri, Wael Sabbah, Roberto Ariel Abeldaño Zuñiga, Ana Vukovic, Julijana Jovanovic, Ro’aa Mohammed Jafar, Ilze Maldupa, Arheiam Arheiam, Fausto M. Mendes, Sergio E. Uribe, María del Carmen López Jordi, Rita S. Villena, Duangporn Duangthip, Nadia A. Sam-Agudu, Maha El Tantawi

**Affiliations:** ^1^ Department of Child Dental Health, Obafemi Awolowo University, Ile-Ife, Nigeria; ^2^ Oral Health Initiative, Nigerian Institute of Medical Research, Yaba, Nigeria; ^3^ Division of Preventive and Restorative Oral Health Sciences, UCLA School of Dentistry, Los Angeles, CA, United States; ^4^ Department of Oral and Maxillofacial Surgery, Obafemi Awolowo University, Ile-Ife, Nigeria; ^5^ Department of Pediatric Dentistry and Dental Public Health, Faculty of Dentistry, Alexandria University, Alexandria, Egypt; ^6^ Department of Dentistry, Muhimbili National Hospital, Dar es Salaam, Tanzania; ^7^ Department of Restorative Dentistry, Muhimbili University of Health and Allied Sciences, Dar es Salaam, Tanzania; ^8^ Department of Community Health and Nutrition, Tanzania Food and Nutrition Centre, Dar es Salaam, Tanzania; ^9^ Department of Paediatric Dentistry, Faculty of Dentistry, University of the Western Cape, Cape Town, South Africa; ^10^ Department of Oral Health, University of Zimbabwe, Harare, Zimbabwe; ^11^ Department of Paediatrics, University of Zimbabwe, Harare, Zimbabwe; ^12^ School of Policy and Global Affairs, Fairleigh Dickinson University, Vancouver, BC, Canada; ^13^ Department of Community Oral Health, School of Dentistry, Tehran University of Medical Sciences, Tehran, Iran; ^14^ Department of Substitutive Dental Sciences, College of Dentistry, Imam Abdurrahman Bin Faisal University, Dammam, Saudi Arabia; ^15^ Guanghua School of Stomatology, Hospital of Stomatology, Sun Yat-sen University, Guangzhou, China; ^16^ Department of Preventive and Community Dentistry, Faculty of Dentistry, Universitas Gadjah Mada, Yogyakarta, Indonesia; ^17^ Department of Endodontics, Faculty of Dentistry, University of Salerno, Salerno, Italy; ^18^ Department of Pediatric and Preventive Dentistry, Kannur Dental College, Anjarakandy, India; ^19^ Department of Orthodontics, Pediatric and Community Dentistry, College of Dental Medicine, University of Sharjah, Sharjah, United Arab Emirates; ^20^ Department of Preventive Dentistry, Faculty of Dentistry, Jordan University of Science and Technology, Irbid, Jordan; ^21^ Department of Public Health and Community Medicine, Jordan University of Science and Technology, Irbid, Jordan; ^22^ College of Dental Medicine, QU Health, Qatar University, Doha, Qatar; ^23^ Faculty of Dentistry, Oral & Craniofacial Sciences, King’s College London, London, United Kingdom; ^24^ Postgraduate Department, University of Sierra Sur, Oaxaca, Mexico; ^25^ Centre for Social Data Science, Faculty of Social Sciences, University of Helsinki, Helsinki, Finland; ^26^ Clinic for Pediatric and Preventive Dentistry, School of Dental Medicine, University of Belgrade, Belgrade, Serbia; ^27^ Department of Pediatric Dentistry, University of Khartoum, Khartoum, Sudan; ^28^ Department of Conservative Dentistry and Oral Health, Riga Stradins University, Riga, Latvia; ^29^ Department of Dental Public Health and Preventive Dentistry, Faculty of Dentistry, University of Benghazi, Benghazi, Libya; ^30^ School of Dentistry, Universidad Austral de Chile, Valdivia, Chile; ^31^ Baltic Biomaterials Centre of Excellence, Headquarters at Riga Technical University, Riga, Latvia; ^32^ School of Dentistry, University of la Republica, Montevideo, Uruguay; ^33^ Department of Pediatric Dentistry, School of Dentistry, University San Martin de Porres, Lima, Perú; ^34^ Faculty of Dentistry, The University of Hong Kong, Hong Kong, Hong Kong SAR China; ^35^ International Research Center of Excellence, Institute of Human Virology Nigeria, Abuja, Nigeria; ^36^ Institute of Human Virology, University of Maryland School of Medicine, Baltimore, MD, United States; ^37^ Department of Paediatrics and Child Health, University of Cape Coast School of Medical Sciences, Cape Coast, Ghana; ^38^ Department of Pediatric Dentistry and Dental Public Health, Faculty of Dentistry, Alexandria University, Alexandria, Egypt

**Keywords:** child abuse, oral health, human rights, legislation, public health

## Abstract

**Background:**

Child neglect is a public health, human rights, and social problem, with potentially devastating and costly consequences. The aim of this study was to: (1) summarize the oral health profile of children across the globe; (2) provide a brief overview of legal instruments that can offer children protection from dental neglect; and (3) discuss the effectiveness of these legal instruments.

**Methods:**

We summarized and highlighted the caries profile and status of implementation of legislation on child dental neglect for 26 countries representing the World Health Organization regions: five countries in Africa (Nigeria, South Africa, Sudan, Tanzania, Zimbabwe), eight in the Americas (Argentina, Brazil, Canada, Chile, Mexico, Peru, Unites States of America, Uruguay), six in the Eastern Mediterranean (Egypt, Iran, Libya, Jordan, Qatar, Saudi Arabia), four in Europe (Italy, Latvia, Serbia, United Kingdom), two in South-East Asia (India and Indonesia) and one country (China) with its special administrative region (Hong Kong) in the Western Pacific.

**Results:**

Twenty-five of the 26 countries have legal instruments to address child neglect. Only two (8.0%) of these 25 countries had specific legal instruments on child dental neglect. Although child neglect laws can be interpreted to establish a case of child dental neglect, the latter may be difficult to establish in countries where governments have not addressed barriers that limit children's access to oral healthcare. Where there are specific legal instruments to address child dental neglect, a supportive social ecosystem has also been built to facilitate children's access to oral healthcare. A supportive legal environment, however, does not seem to confer extra protection against risks for untreated dental caries.

**Conclusions:**

The institution of specific country-level legislation on child dental neglect may not significantly reduce the national prevalence of untreated caries in children. It, however, increases the prospect for building a social ecosystem that may reduce the risk of untreated caries at the individual level. Social ecosystems to mitigate child dental neglect can be built when there is specific legislation against child dental neglect. It may be more effective to combine public health and human rights-based approaches, inclusive of an efficient criminal justice system to deal with child dental neglect.

## Introduction

Child neglect is a public health, human rights, and social problem, with potentially devastating and costly consequences (1). Globally, it is estimated that up to one billion children aged 2–17 years have experienced physical, sexual, or emotional violence or neglect in the previous 12 months (2). The actions or inactions of parents, caregivers and other authority figures resulting in the neglect of children and adolescents are classified as maltreatment (3). Child maltreatment or neglect result in neurological and biological events that have a negative impact on physical and behavioral health and have academic and economic consequences (4).

One such physical health consequence of child maltreatment or neglect is poor oral health. Oral health is vital for the overall health and well-being of children (5), and it is recommended that they have annual dental visits to receive oral screening and risk assessment with timely and comprehensive preventive dental care planned according to assessed risk (6). Children with oral diseases also need dental clinic visits for disease management to ensure freedom from pain and infection (7). Caries is an important oral disease; untreated caries in primary teeth and permanent teeth affect 621 million children and 2.4 billion people, respectively, worldwide (8). Untreated caries negatively impacts quality of life for children, affecting their ability to learn, play, eat and sleep. Severe caries can contribute to failure to thrive and limits children's ability to live a fulfilling and productive life (9–15). Parents and other caregivers are guilty of dental neglect when they fail to pursue the dental care required to maintain their child's oral health (7).

Dental health professionals in countries like the United Kingdom are required to ensure that all children and adolescents have access to preventive dental care and treatment for oral disease and injury, to mitigate the risk of dental neglect (16). This is important, because a case of dental neglect can be made if a parent persistently fails to obtain treatment for their child's dental caries when such dental services are available (17). While there is no evidence to suggest a specific threshold of oral disease to diagnose dental neglect, a case of dental neglect can be made when there are features of a lack of basic oral care, including oral hygiene, failure to seek treatment for oral pain, and leaving infections untreated (17).

Neglect is often dictated by culture [ways of thinking, feeling and behavior that are socially and not biologically transmitted from one generation to the next (18)], tradition, religion and familial norms that influence home life, parenting practices, and upbringing. This makes what is considered neglect highly context-specific and often controversial, with socio-economic status adding another layer of complexity. While the influence of culture on child-rearing practices has been well studied (19, 20), there is little known about how cultural nuances may influence perceptions of child neglect and its impact on child oral health. Irrespective of cultural nuances, official recognition of dental neglect as a form of child neglect raises the profile of child oral health in the public health agenda (16), potentially resulting in the institution of legal instruments to reduce the risk of child dental neglect.

The aim of this study was to: (1) summarize the oral health profile of children in different countries across the globe; (2) provide a brief overview of existing legal instruments that can offer (or have offered) protection for children from dental neglect; (3) examine limitations on implementation of these legal instruments, which criminalize child dental neglect; and (4) discuss facilitating actions to reduce the risk of children's exposure to dental neglect.

## Methods

This narrative review presents country-level status quos regarding child dental neglect, using series of case studies with varying contextual backgrounds. For this study, we broadly define policy as government actions in response to societal issues, specifically focusing on child dental neglect and poor oral health in children. These policies may take the form of regulations, rules, or administrative codes with the force of law, and may be based on statutory interpretations (21, 22). We also recognize that policies pertaining to child neglect serve as social (protective) regulatory guidelines that establish boundaries for permissible behavior within their jurisdictions (23). We centered our attention on the policy analysis stage of the policy-making process, as outlined by Dunn (24) and Howlett and Ramlesh (25). The objective was to assess policies, identifying any potential issues, to provide further guidance (26).

To achieve this, we have compiled a summary of legislations on child dental neglect from 26 countries representing the World Health Organization (WHO) regions: five countries in Africa (Nigeria, South Africa, Sudan, Tanzania, Zimbabwe), eight in the Americas (Argentina, Brazil, Canada, Chile, Mexico, Peru, Unites States of America, Uruguay), six in the Eastern Mediterranean (Egypt, Iran, Libya, Jordan, Qatar, Saudi Arabia), four in Europe (Italy, Latvia, Serbia, United Kingdom), two in South-East Asia (India and Indonesia) and one country and its special administrative region in the Western Pacific (China and Hong Kong). We adopted a case-study method of policy analysis (27), which allows the contextualization of problem definition and sheds light on issues relevant to policy-setting based on real life conditions. By presenting case studies from countries with different cultural, economic, social, and healthcare backgrounds, we highlight contrasting approaches to address the same problem: child dental neglect. Although the available literature about the extent of dental neglect and relevant policies is too sparse for hypothesis generation, we theorize that countries with developed health and social care systems have well-defined policies to address child dental neglect. This theory guides the selection of case studies from countries in all WHO regions since it is the global authority on healthcare policy recommendations.

The summary of legislations on child dental neglect for each country was compiled by conveniently selected country experts who understood both the legal context of child dental abuse and the magnitude of oral health problems in their respective countries. The primary consideration for selecting each country expert was a history of publication on country-specific oral health problems. Each country expert was required to conduct a search of databases (PubMed, Scopus, PsycINFO, and CINAHL) for studies and documents on the prevalence of oral diseases (caries, periodontal diseases) and caries risk behaviors (oral hygiene status and history of dental service utilization), and on child neglect and child dental neglect. A search of grey literature was also conducted. The findings were synthesized and summarized into a table. The summary for each country was organized into their respective WHO regions (the Americas, Eastern Mediterranean, African, European, South East Asian, and the Western Pacific regions).

The commonalities and differences within and between regions were reported. The links between the presence (or absence) of policies and the prevalence of oral diseases (specifically dental caries) was addressed. Dental caries was selected because it is the only childhood oral disease with globally available data that allows for standardized between inter-country comparison. The dental caries analysis was based on the Global Burden of Disease studies used in the 2022 WHO Global Oral Health Status Report. Findings were discussed, and proposals were made for policy changes to effectively address child dental neglect.

## Results

### African region

#### Nigeria

In Nigeria, the burden of untreated caries in children is a cause for concern, as the prevalence of untreated caries is high (28). The prevalence of poor oral hygiene ranges from 0.2% in 1–5-year-olds to 4.4% in 6–12-year-olds (29); and for gingivitis ranges from 63.3% in 6–11-year-olds (30) to 57.2% in 10–19-year-olds (31). Untreated caries in children is over 80% and results from poor uptake of oral health services for several reasons, ranging from high out-of-pocket expenditure and poor insurance coverage for oral healthcare (32), low socioeconomic status (33), low level of maternal education (34), perceiving hospital visits as synonymous with need for curative care (35), poor perception of the need for oral health care (36), to rurality and a complex oral health system that is difficult to navigate (37).

The 2003 Child's Right Act guarantees the rights of children in Nigeria (38), and the National Human Rights Commission undertakes actions to promote and protect the rights of children from fetus to 17-years of age (39). Section 2 (1 and 2), and Section 13 (1 and 2) of the Act recognize the right of children to health. Section 13 (5a and 5b) place the liability of child oral health care on caregivers, punishable with a fine for the first offence and a one-month imprisonment for a second offence. The Act does not explicitly mention child dental neglect as a criminal offence.

Section 13 subsections 3b and 3g, however, create a leeway to exonerate caregivers from an offence of child (dental) neglect by indicating that the government has the responsibility to provide medical assistance and health care services to all children through the development of primary health care services. However, the government only provides dental care through 27 (0.08%) of the 33,156 primary health care centers in Nigeria (40, 41), which indicates severely limited access to dental care in the country. In addition, dental care is expensive (42), health insurance coverage is low and extremely limited for oral health care-almost to the exclusion of preventive oral health care services (37)- and human resources for oral health care are scarce (42, 43). These gaps make it difficult to establish a charge of child dental neglect in Nigeria. In addition, most dentists in Nigeria do not screen for dental neglect (44), only 51.3% recognize neglect as a form of child abuse (45), and only 14.1% of those who suspect child abuse and neglect file reports to social services (44). To date, there has been no legal prosecution of a case of child dental neglect in Nigeria.

#### South Africa

Early Childhood Caries (ECC) is a significant public health problem in South Africa, with prevalence reaching 60% in 2004, which is the most recent estimate (46). The prevalence of ECC had increased to 71.6% in 2018 for children resident in low socioeconomic communities, of whom 70% need dental treatment (47). Parents are responsible for their child's basic needs, including diet, personal hygiene, and access to health care, and therefore play an important role in preventing dental disease in their children. The high rates of untreated caries and extractions may be pointers to child neglect, though this needs to be contextualized.

South Africa has a population of approximately 60 million people, of whom 9.4% are 5 years and younger, and 28% are 15 years and younger (48). The South African population is diverse in socioeconomic status, living conditions, access to health care and quality education, and therefore, diverse in caries risk (49). Although 71.9% of households access public clinics, only 16.1% have medical aid (50). Limited access to oral health care results in high prevalence of untreated caries with many children requiring multiple extractions under dental general anesthesia (51).

Dental practitioners are trained to be sensitive towards inequalities and to differentiate between ignorance, lack of education, limited access to oral health care and dental neglect. Section 110 (1) of the Children's Act (No 38 of 2005) (52) states that if there is a reasonable suspicion (on the part of treating professionals) that a child is being abused in a way that causes physical injury, sexual abuse or neglect, this must be reported. A range of professions including legal practitioners, medical and dental practitioners, traditional and religious leaders, psychologists, social workers, teachers and persons working in care facilities, are empowered to report neglect to a social worker, a designated child protection unit or organization or to the police [Section 110 (2)]. The Children's Amendment Act (2007) (53) is more detailed and provides psychosocial, rehabilitation and therapeutic services for neglected children. The South African law is, however, not specific on child dental neglect.

#### Sudan

There are 17 states in Sudan. However, data about the prevalence of dental caries, and decayed, missing, and filled teeth (DMFT) values are available only in the three states of Khartoum, Kassala and Gazeerah. The prevalence of caries in children and adolescents is very high, and ranges from 70.9% (54) to 91.1% (55).

The Sudanese's Child Act (2010) (56) is housed by the Ministry of Justice and addresses the rights of children with respect to nutrition and breastfeeding, immunization, physical and mental health, and protection of children from infectious diseases and physical and sexual exploitation. There are also chapters on the right of children to education, and the rights of vulnerable children. However, to date, there is no legislation to protect the Sudanese child from dental neglect. There are currently no studies investigating the prevalence, knowledge, causes and types of dental neglect in Sudan.

#### Tanzania

In Tanzania, more than 3 in 10 children have experienced dental caries (57), and 5-year-old children have higher dental caries experience than 12 and 15-year-olds, with the decay component accounting for 99% of the total caries experience (57). Also, 57.4% of 12 and 15-year-olds have gingivitis and 4.4% have experienced dental trauma, with more children affected in rural than urban areas (65.2% vs. 52.0% and 5.7% vs. 2.7% respectively) (57).

Tanzania is a signatory to the 1989 Convention on the Rights of the Child and the 1990 African Charter on the Rights and Welfare of the Child. In 2009, Tanzania enacted the Law of the Child Act to accommodate the requirements of International Legal Frameworks on the protection of children (58). Section 4 (2) of the Act insists that the best interest of a child should be the primary consideration in all actions taken with respect to children. Section 9 (1) provides that parents, guardians and relatives are responsible for ensuring children's right to life, dignity, respect, leisure, liberty and health. Section 13 (1) prohibits degrading treatment to children, and institutions like local government authorities and police are vested with the legal duty to ensure children's safety. Despite the enactment of the Law of the Child Act, child neglect is still a serious problem in Tanzania (57). The 2020 UNICEF report noted that many children in Tanzania experience violence, neglect, and exploitation (59). There is no enforcement mechanism to monitor children's protection.

Efforts to strengthen the 2009 Child Act have not been sufficient to address dental neglect in children. Strategies to improve the knowledge and awareness of caregivers toward children's daily oral health practices and correct feeding habits are essential in addressing dental neglect in Tanzania (60). Furthermore, tailored public education programs addressing dental neglect and building the competency of oral health personnel to detect and manage dental neglect are needed.

#### Zimbabwe

The caries prevalence of 12-year-olds has increased from 27.6% in urban areas and 20.9% in rural areas in 1989 (61), to 59.5% among urban and 40.8% among rural children in 2013 (62). Furthermore, 65.2% of carious teeth are untreated and 34.8% are missing (63). The instituted school-based oral health programs responsible for tackling oral health problems in children attending primary and secondary schools (64) have limited effectiveness (65). Many children present with untreated carious lesions that their parents have the responsibility to manage.

Zimbabwe adopted the Children's Act (Chapter 5:06) in 2001 to domesticate several international treaties on childcare and protection it was a signatory to. The Act states that a parent or guardian of a child or young person shall be deemed to have abandoned or neglected that child or young person if (s)he …(b) failed to provide or pay for dental, medical, or surgical aid or other effective remedial care necessary for his/her health or well-being. The Act is enforced by the Victim Friendly Unit of the Zimbabwe Republic Police which works closely with hospital police posts or police stations to handle reported cases of child abuse. Currently, there is no stand-alone legislation on child dental neglect.

The effectiveness of the Children's Act to deal with child dental neglect is currently limited, as there are no defined parameters to identify cases of child dental neglect in the country. No parameters that define child dental neglect and abuse have been declared to the public and health service providers, and cases remain undetected and unassisted. In addition, the curriculum of undergraduate and postgraduate dental students does not cover this aspect of childcare.

### Region of the Americas

#### Argentina

In 1985, 40% of children aged 7% and 68% of children aged 13 years had caries in permanent teeth (66). By 2010, caries was reported in 49% of 4-year-old children, of which only 10% had treatment (67). In 2019, 70% of children had caries (68, 69).

Argentina ratified the International Convention on the Rights of the Child in 1990. The International Convention on the Rights of the Child led to the incorporation of Article 75 into the new Constitution of the Argentine Nation, and the 2005 National Law 26,061 on Comprehensive Protection of the Rights of Girls, Boys and Adolescents. The 2005 National Law establishes that “*Girls, boys and adolescents have the right to comprehensive health care, to receive the necessary medical assistance and to have equal access to opportunities for services and actions for prevention, promotion, information, protection, early diagnosis, timely treatment and recovery of health”* (70). In 2017, the Oral Prevention and Primary Assistance Program was created, through Resolution 440-E/2017 of the Ministry of Health, but it does not have the force of Law.

Greater emphasis is placed on addressing the increasing violence in schools, societal fragmentation, and inequalities than on addressing oral health problems. This leads to the neglect of oral health (71), which, if addressed, should reduce the burden of oral diseases in children. However, children's oral health continues to be a public health problem despite the presence of relevant legislation, and epidemiologic indicators reflect this.

#### Brazil

Preliminary results obtained from 25% of the required sample of the ongoing Brazilian Oral Health Epidemiological Survey indicates that prevalence of caries in the permanent dentition is 49.2% and 50.7% in the primary dentition (72). Also, the mean number of teeth with untreated caries was 1.82 for 5-year-olds and 1.04 for 12-year-olds (72). The prevalence of caries in the permanent dentition has decreased over the years. However, this same trend has not been observed for primary dentition.

Child neglect is addressed through the Child and Adolescent Statute (ECA—Estatuto da Criança e do Adolescente), a federal law created in 1990 to establish the right and protections of Brazilian children and adolescents (73). The ECA states that the Brazilian public health system (SUS—Sistema Único de Saúde) must promote general and oral health care programs focusing on the most prevalent diseases affecting children (73). The ECA also notes that the family, community, and government should ensure that children have access to oral health care. Oral health care should be promoted for pregnant women and children through education and prevention before the child's birth, and when the children are 6 and 12 years of age. Furthermore, the cost of treatment for children with dental treatment needs should be covered by the SUS.

In 2004, the Brazilian Policy of Oral Health was created and promoted the reorganization of primary health care by expanding oral health teams, the family health strategy and creating dental specialty centers (74). However, the program has been criticized due to the poor coverage of children with oral health needs, emphasis on operative treatment, and lack of trained professionals, mainly for children requiring complex dental treatments. These problems can be ascribed to the low utilization of dental services: only 25% of preschool children make use of these services (75). Dental caries indicators for primary teeth have not improved in the last decade (72, 76, 77).

#### Canada

Untreated dental caries and untreated dental trauma are two examples of reportable dental neglect among children in Canada (78). The 2007–2009 National Oral Health Survey indicated that 56.8% of 6–11-year-old Canadian children had dental caries, with untreated carious lesions accounting for 14.7% of all reported cases (79). Low-income (17.6%) and uninsured families (19.1%), those who had not visited a dentist in the previous 12 months (30.9%), and families with low levels of education (18.9%) had more untreated caries than better-off subgroups with various socio-demographic characteristics (79). Among adolescents, caries prevalence was 59.8%, the proportion of untreated caries was very low, with higher prevalence among those from lower, than higher income groups by more than threefold (80). Among children of Indigenous descent, nearly half of the caries lesions in preschool children were untreated (80). The reasons caregivers delay dental treatment are mainly because of their inability to pay for dental care and the perception that primary teeth will eventually be lost (81).

When a parent fails to act after being properly informed about the nature of their child's oral health problem and the specific treatment required, this is considered negligence and requires intervention. There are laws at the federal and provincial levels in Canada that provide the legal framework to address child dental neglect. The law requires that dentists report dental neglect only after questioning the child and the parent separately about the clinical presentation, with a staff member present to act as a witness (82).

There are several agencies in Canada that can receive and investigate reports of dental neglect. These include the local Royal Canadian Mounted Police, municipal police station, and the government ministry in charge of child and family welfare. Provincial and territorial legislations have been helpful in early detection and intervention for child neglect in Canada (83). However, there is little evidence to assess their effectiveness in addressing child dental neglect.

#### Chile

Chile has a favorable children's rights environment, scoring 9.22 out of 10 and receiving a “green” classification (84). The estimated prevalence of untreated caries in the primary dentition among Chilean children aged 1–9 years is 46.6% (85), with a higher prevalence in rural areas (86). Also, the prevalence of dental trauma [37.95% (87)] and malocclusions [38%–53% (88),] are high.

Chile's does not have a specific law focusing on comprehensive child rights protection. Instead, it relies on the Intersectoral System of Social Protection (Chile Solidario), which includes the Chile Grows with You (CGY, Chile Crece Contigo) program (89). Established in 2006, CGY is a comprehensive program that supports early childhood education, child health and nutrition services, family support, and social protection through coordination between various ministries and institutions, such as the Ministry of Social Development and Family, the Ministry of Health, the Ministry of Education, and the National Board of Kindergartens (89). It incorporates an oral health component called “Oral Health in Your Life” (Salud Oral en tu Vida), promoting good oral health habits among pregnant women, infants, and children up to four years old through education and preventive services (90). The creation of the Children's Ombudsman Office (Defensoría de la Niñez) in 2018 strengthened Chile's commitment to safeguarding children's rights (90).

In addition, Chile has implemented several policies using a common risk approach to address sugar consumption, which contributes to dental neglect. These policies include a tax on sugary drinks containing more than 6.25 g of sugar per 100 ml (91). The allowable amount of sugar in foods without warning labels has also been reduced from 22.5 g per 100 g to 10 g per 100 g. In 2016, the Law of Food Labeling and Advertising was enacted. This requires front-of-package warning labels, restricts child-directed marketing, and bans sales in schools of all foods and beverages containing added sugars, sodium, or saturated fats that exceed set nutrient or calorie thresholds (91). The government also covers the cost for essential curative (but not preventive) oral health care for six-year-old children through the Explicit Health Guarantees. This is, however, a counterproductive program as children already have dental caries at a much younger age and need care much earlier: 80% of two-year-olds have dental caries, and the provision of oral health care at a later age does not align with the strategies of the Oral Health Department of the Chilean Health Ministry which aims to have at least, 80% are children been caries-free (92). These actions are all together complex and make it difficult to establish cases of child dental neglect in Chile.

#### Mexico

Caries has been a growing problem over the last 30 years in Mexico, with the current prevalence of caries estimated to range between 80% and 90%, and at least 10 million children between 2 and 5 years of age having caries (93, 94).

The Convention on the Rights of the Child (95), the American Convention on Human Rights (96) and the General Law on Human Rights of Girls, Boys and Adolescents (97) establishes the obligation of the Mexican State to guarantee the right to health of people younger than 18 years of age. This broad legal framework recognizes three guiding principles that the Mexican state must consider in relevant actions: non-discrimination, superior interest, and recognition of evolutionary capacities, in accordance with their interests and needs. Likewise, the General Health Law establishes the right to protect the health of the population, through actions to promote health, disease prevention and control, among others (98). Neglect is criminalized through the Comprehensive Care Clinic for Abused Children of the National Institute of Paediatrics that defines child abuse as “*any aggression or intentional omission, whether physical, sexual, psychological, or negligent, within or outside the home, against a minor, before or after birth, that affects their biopsychosocial integrity, regularly or occasionally carried out by a person, institution or society, based on their physical, intellectual or economic superiority”* (99). Parents and children's guardians are responsible for ensuring the health of their children or wards. However, Mexico is a country of profound social inequalities, with 28.6% of children never having contact with a dentist. This poor dental service utilization is associated with indicators of socioeconomic status and reflects inequality in dental service access (100, 101). The challenges that government face in addressing inequality in dental service access makes the case of child dental neglect difficult to establish in Mexico.

#### Peru

Peru is promoting a reform process that supports achieving universal health coverage. Since 2015, health insurance enables pregnant women and children up to five years of age to access free health care. When there is no health insurance, the newborn is automatically affiliated with the Comprehensive Health Insurance (SIS) regardless of their socioeconomic status.

The last national study (2001–2002) of the Ministry of Health that was carried out in children 3 to 15 years old showed a prevalence of dental caries ranging from 57.6% to 63.5% with a mean of 60.5% in the primary dentition, and from 56.2 to 65.1% with an mean of 60.6% in the permanent dentition. 10 years later, an unpublished 2012–2014 national study conducted by the National Epidemiology Center of the Ministry of Health found very similar values without significant differences in both dentitions. The data is similar to that reported for the Americas by the WHO in 2022 (102, 103).

In 2017, a Ministerial Decree modified the Technical Health Standard for the Control of Growth and Development of children under 5 years of age. The modification included a provision that the health professional (nurse, pediatrician, or general practitioner), would review the child's mouth by age one year, and all children should be checked by a dentist before one year of age. However, there is no national dental program with well-defined guidelines for monitoring child oral health. Also, although Peruvian law 30364 (104) promotes protection against violence affecting women and children, there is no specific rule that recognizes poor child oral health as an act of neglect. There is also low uptake of dental health services in the population under 12 years of age (105), and a lack of training and integration of child oral care into multidisciplinary practices. There are however good intentions to address these gaps in order to achieve established universal health goals.

#### United States of America

Although completely preventable, ECC is currently the most common chronic disease in children from the United States of America, and its prevalence continues to be high in poor and near-poor children of preschool age (106, 107). Furthermore, socially disadvantaged children are less likely to have access to oral health care, instead seeking care for oral infections from emergency departments and hospitals (108).

Parents must be informed by a health care provider about the necessity of oral health care for their child, their child's oral health status, the treatment required, and how to access that treatment (109). Since some caregivers may, however, be unaware of their children's need for dental care, it is important to differentiate between a lack of oral health literacy and willful failure to seek care when determining whether a situation qualifies as dental neglect (110). Also, there are several barriers that may interfere with parents seeking appropriate oral health care for their child (110–112). If a provider has ascertained that the parent understands what dental care their child needs and how to obtain it, and the above factors still indicate a case of dental neglect, the provider should report the case to the appropriate child protection services agency (109).

It is important that health care providers from all disciplines are aware of signs that indicate child abuse and/or neglect (109). The American Association of Pediatric Dentistry states that all health care providers, including dental providers. are expected according to local or state requirements to report injuries that cause concern for abuse or neglect to child protective services (108). Additionally, they are encouraged to collaborate with interprofessional colleagues to provide support for affected families (109).

#### Uruguay

The prevalence of dental caries is high, and worse among those with low socioeconomic status: in the poorest populations the rate of caries disease is five to eight times higher. The DMFT among 12-year-olds is 4.2–4.7 in the most deprived areas, and 2.5 among those with high socioeconomic status (113). Among adolescents, 60% of those with high socioeconomic status are caries-free, while only 11% of those with low socio-economic status are (114). A study conducted in 2017 (115) indicated that most children have active untreated cavitated lesions, possibly due to poor access to timely care, and that if the approximately 25% of 5-year-old children with non-cavitated lesions could have access to early treatment, cavitation, and complex and expensive treatments could be avoided.

The 2004 law 17.823 on children and adolescents (116) is applied through the National Integrated Health (SNIS) law 18.121 of December 2007, and implemented by the Ministry of Public Health ((117). The law articulates that public and private providers of comprehensive health care should ensure access to comprehensive health services for all residents in the country. Uruguay has a National School Oral Health Plan under the State Health Services Administration (ASSE-MSP) that serves around 75,000 children per year and develops a program for the prevention and minimal intervention with 430 dentists and two mobile clinics that operate throughout the country. However, the integration of dentistry into the general health system could be better, as that may facilitate detecting and addressing child dental neglect.

### Eastern Mediterranean

#### Egypt

A 2014 nationwide survey indicated that nearly 70% of Egyptian children had untreated caries and about 80% had periodontal disease (118). In 2009, Egypt developed a national strategy to combat abuse, including activating laws, raising awareness, and establishing agencies to help victims of neglect (119). This was in response to articles 80 and 96 of the Egyptian constitution, which stipulate that the state shall care for children and protect them from all forms of neglect threatening their wellbeing (120). Alhough the Egyptian Law penalizes child neglect after receiving a warning notice (120, 121), the law does not criminalize the parental negligence of children.

The National Council for Childhood and Motherhood has a helpline for child complaints, and it provides counseling and referral services to victims of violence, abuse, and neglect. The Ministries of Health, Social Services and Interior, and non-governmental organizations are responsible for child protection (122). However, reports of neglect is low, due to poor public awareness of reporting mechanisms (123). In 2022, only 13.6% of citizens were aware of the presence of agencies supporting abuse victims, and 7.7% were aware of a hotline to report instances of neglect (124). Though dentists can play a role in diagnosing and managing child dental neglect, only 40% of dentists indicated ever suspecting abuse in their patients, despite learning about social indicators of child maltreatment in dental school (123, 125).

There is no law that mandates reporting of child maltreatment, and there is no penalty for non-reporting. Currently, only schoolchildren 6 to 18 years old have access to regular care covered by health insurance, and it is expected that this coverage will extend to preschool children when the Universal Health Insurance Law is fully applied at a national level (126). The persistence of untreated oral diseases in children with health insurance coverage and access to regular care should indicate dental neglect. However, limited parental literacy and poor oral health values (60) reduce the possibility that parents would be charged for dental neglect of children. In addition, no laws or guidelines specifically identify cases of dental neglect, and there is a paucity of information about the prevalence of child dental neglect.

#### Iran

The prevalence of dental caries in primary teeth among children in Iran has been on the increase in the last 28 years (127). The dmft index for 5–6-year-old children is 5.16, and 86.9% of it is related to the decay component. The DMFT index in 12-year-old children is 2.1, and 81.8% of this index is related to decayed teeth (127, 128). In Iran, child neglect is considered a social emergency (129). The Iranian welfare organization has specifically targeted children living on the streets, children who are working, and children who are abused and neglected, for priority interventions. The public is expected to place a call to the Social Emergency Department of Welfare (Behzisty) organization if anyone identifies a case of child abuse or neglect.

Article 4 of the Law on Protection of Children and Adolescents was approved in 2003. It states that any physical and mental harm or torture of children and neglect deliberately affecting their mental and physical health or preventing them from studying, is prohibited and punishable by imprisonment or a monetary fine. Also, Article 1173 of the Civil Law (amended 1998) can be invoked in case of non-observance of the child's physical health by parents (130). The Article states that when the physical health of the child is in danger due to a lack of care or moral degeneration of the father or mother under whose custody the child is, the court can take any decision deemed appropriate for the custody of the child upon the request of the child's relatives, guardian or the head of the jurisdiction.

Despite the law, only advanced and life-threatening mental or physical health problems are reported, with very low public sensitivity to dental neglect. The absence of local guidelines on child dental neglect has also complicated matters (131). The faculties in the School of Dentistry, Isfahan University of Medical Sciences and the Ministry of Health are developing evidence-based guidelines for the reporting of child abuse and neglect, including the reporting of child dental neglect by dentists. These guidelines will take a lot of resource investment to implement throughout the country. It is, however, a welcome development to complement the existing law for legally addressing child dental neglect in Iran.

#### Libya

The oral health care system is hybrid, composed of both public and private sectors. Although health care policy in Libya implies that free care should be provided to all, only few emergency services are provided in the public sector, and most of dental services are provided as fee-per-service in the private sector. Several studies have demonstrated highly unmet treatment needs among school children: 70% of 6-year-olds and 42% of 12-year-olds have untreated caries (132, 133). There is also a high level of unmet treatment needs related to dental trauma (134), dental fluorosis (135) and molar incisor hypo-mineralization (136).

Although professionalism and ethics are key subjects in the dental curricula in Libya, anecdotal evidence indicates that dental neglect does not receive much attention compared to other medicolegal topics. The Libyan law has clear legislation and guidance on how to deal with professional neglect that causes morbidity and mortality. However, there is no clear legislation that describes what should be done to protect children from domestic child neglect. There is also no national guidance on protecting the rights of children with dental neglect or other forms of health neglect. This calls for advocacy for these issues in Libya, in addition to strengthening dental curricula to build competency on the identification and management of dental neglect.

#### Jordan

Research on child dental neglect in Jordan is scarce. Parents have poor knowledge about their children's oral health, and parental opinion of their children's oral health status s overrated (137). There are currently no country-level legal instruments that specifically address child dental neglect in Jordan. However, children in Jordan have specific rights under the United Nations Convention on the Rights of the Child, to which Jordan is a signatory (138). In addition, the Jordanian constitution guarantees the right of the child to protection and the Draft Jordanian Child Rights Law provides comprehensive protection for the rights and well-being of all children residing in the country (139).

In addition, the government provides health insurance, which covers dental treatment for children under age six years (140). In 1991, Jordan ratified the Convention on the Rights of the Child (141). In 2011, the country added two new paragraphs to Article 6 of its Constitution, which state that “*the law shall protect motherhood, childhood and the old-aged; and shall avail care for the youngsters and those with disabilities and protect them against abuse and exploitation”* (142). Despite the existence of such legal instruments, there are limitations with their implementation, such as poor awareness of parental legal obligations to provide adequate dental care for children, limited access to dental care particularly in rural areas, lack of law enforcement systems to hold parents responsible for dental neglect, poor parental prioritization of dental care, and financial barriers. Therefore, it is necessary to increase awareness and legal obligations of parents to provide dental care through public education campaigns and by working with healthcare providers to promote oral health.

#### Qatar

The State of Qatar has a population of 2.7 million and has the six-highest GDP per capita in the world at the end of 2022 (https://www.visualcapitalist.com/cp/ranked-countries-gdp-per-capita-2023/). A significant shift in lifestyle and dietary habits due to rapid socioeconomic development has led to a marked increase in the prevalence of dental diseases, mainly dental caries, among children. The prevention, oral health promotion, early detection and treatment of dental diseases had been largely driven by the Primary Health Care Corporation that target high-risk groups, including pregnant women, infants, and school children. Oral health screening is provided to kindergarten and schoolchildren through the “Asnani School Oral Health Program”. Despite these efforts, the prevalence of dental caries among children is still high: 85% of 12–14-year-olds have caries, with the mean number of decayed teeth being 4.62 (143); 71.4% of 6-year-olds have caries with a mean dmft of 4.2 (144); and 89.2% of 4–5-year-olds have caries with dmft of 7.6 and dt of 7.1 (145). These findings suggest the presence of dental neglect and unsatisfactory oral health status.

Qatar adopted legislation to ensure child protection and to prevent abuse (146). The Qatari constitution states that: “*The State shall provide care for the young and protect them from corruption, exploitation, and the evils of physical, mental and spiritual neglect”* (147). The first child abuse protection program, the Sidra Child Advocacy Program (S-CAP), was established by Sidra Medicine to provide child abuse services by well-trained pediatricians and supporting staff. S-CAP includes safe, discreet facilities for conducting interviews, medical examinations, and creating medico-legal reports. Additionally, S-CAP established a child hotline for the public to report any suspected child abuse (148).

There are, however, no specific legal provisions related to child dental neglect. The extension of the current laws on child protection and the inclusion of child dental neglect in the mandate of S-CAP may further promote oral health among children, reduce the burden of dental disease and positively impact the wellbeing and quality of life of children in Qatar. The identification and reporting of dental neglect may, however, be difficult, due to challenges with identifying children who are dentally neglected (149), gaps in the knowledge of dentists regarding dental neglect, and the absence of a system for reporting child dental neglect.

#### The Kingdom of Saudi Arabia

The prevalence of dental caries in children has increased significantly, to almost 70% in the permanent dentition and 80% in the primary dentition (150, 151). The prevalence of complicated untreated dental caries among children is high, with up to 4 in 10 children visiting the dental clinic due to tooth ache resulting from caries (151, 152). Although dental care services are free, the prevalence of child dental neglect is high. Hospital-based reports of child abuse rose from 65 cases in 2008 to 73 cases in 2009, 80 cases in 2010 and 616 in 2011 (153). The reasons for this include low levels of parental education (154), poor perception of the need for oral health care, fear of dental treatment, lack of public transportation (155) and inability to obtain a dental appointment because of the long waiting lists (156). Also, working mothers may not have free time to visit the dental hospitals except for emergency care (157).

In Saudi Arabia, child neglect is an offense that must be reported. In August 2013, the law of protection from child abuse and neglect was enacted (158). The Ministry of Education also launched an awareness campaign online to provide information on early childhood care (159). As a part of this campaign, the Child Protection System, the policy of child protection against abuse or neglect and the ways of managing reports submitted to authorities were explained in detail (160). Dentists are responsible for identifying and reporting suspected cases of dental neglect to authorities (161). The police station is the certified agency for handling reports of child abuse and neglect. Police then refer certified cases to child protection teams to manage (158).

Despite the law, the reporting of child dental neglect by dentists is low, due to a lack of training (162). The lack of awareness about the referral process and responsible legal authorities reduces the number of reported cases (163). The competency of dentists to identify cases can be addressed by building additional training elements into the dental curricula to train students on identifying the following: signs and symptoms of neglect, documentation and reporting, the Saudi child protection policy, and the consequences of failure to report abuse and neglect for the child's oral and physical health, and behavioral and psychological development. Training has increased the reporting of child abuse and neglect cases by dentists in Saudi Arabia (164).

### European region

#### Italy

Caries prevalence among 4-year-olds is 21.6% and 43.1% for 12-year-old children in Italy (165). In 2006, approximately 75% of 4-year-olds and 50% of 12-year-olds were reported to have more than one carious lesion, thus, requiring dental care (166).

Italy adopted a legislation to ensure child protection and abuse prevention. Article 572–591 of the Italian penal code states that: “*Anyone who abandons and mistreats a person under the age of fourteen, or a person who is incapable, due to mental or physical illness, of providing for himself, and for whom he has custody or has to take care of, is punished with imprisonment from six months to 24 years*.*”* The Office of the Guarantor Authority for Childhood and Adolescence oversees child projects and reported in its 2018–2020 investigation of 250 out of the 6,080 municipalities in Italy that out of every 1,000 resident minors, 45 were in the care of Social Services. Of the 401,766 children and young people in the care of Social Services, 77,493 (19.3%) had experienced some form of abuse. Twenty percent of these reported cases of abuse came from the hospital, the dentist, and the pediatrician (167).

There is no specific legislation or guideline on child dental neglect, nor are there screenings, reporting or management of child dental neglect in Italy. The 2018–2020 survey highlighted the need for instituting periodic monitoring systems to support the implementation of policies on prevention, protection, and treatment of abused minors (167). However, the current policy supporting children who survive child abuse seems not to have effectively addressed child dental neglect as a distinct form of child abuse despite evidence that child dental neglect is an indicator of child abuse.

#### Latvia

The estimated prevalence of untreated caries in primary teeth is 47.5%, and the prevalence of caries in the enamel level reaches 98.5% in 12-year-olds at the enamel level (168). In addition, the number of cases of dental treatments for children under general anesthesia has increased by 350% over the past 16 years (169). However, despite legislation mandating public dental care for children under 18 years of age, access to dental care is limited due to funding shortages and the need for dentists, particularly in regions outside the capital city.

Latvian laws emphasize the importance of ensuring children's access to healthcare, including dental care, and hold parents or guardians responsible for their child's health and well-being. Neglect is defined as the failure or negligent performance of duties of care and supervision or prolonged neglect that harms the child's development or causes physical or psycho-emotional suffering, as stated in Article 13 of the Law on the Protection of the Rights of the Child (170). Liability for failure to fulfil childcare obligations can result in a warning or a fine, as described in Article 51 (170). Parental custody may also be suspended in cases where the parent endangers the child's health or life, abuses their rights, or fails to ensure the care and supervision of the child, as per Article 200 of the Civil Code (171). However, it is challenging to implement legal instruments to address child dental neglect in Latvia. This is because of limited access to public service, which has resulted in delays in adequate care, a lack of timely interventions and no prevention programs.

To effectively address the oral health needs of children in Latvia, it is imperative to shift towards evidence-based interventions supported by reforms in the payment systems. The adoption of value-based healthcare models (172), which prioritize health outcomes rather than paying for manipulations, could raise public awareness about parents' responsibilities. By implementing these reforms, Latvia can strengthen the application of legal instruments, ultimately leading to improved oral health outcomes for children and reducing the prevalence of caries.

#### Serbia

One third (36.7%) of children aged 12 to 71 months in Serbia had ECC, and a mean dmft of 0.9, with 94.1% of the lesions untreated (173). About 44.3% of parents are actively involved with daily toothbrushing for their children. Children living in poor regions of the country have a higher likelihood of ECC and untreated lesions (173).

Although there is universal dental health care coverage for children and adolescents until age 18 years and for undergraduate students until age 26 years, there is poor uptake of the free public dental services: 23% of infants received oral health examination and advice before their first birthday, 18% of parents of infants received free individual oral health education, 20% of toddlers received oral health examination, 17% of parents of toddlers received free individual oral health education and 20% of 3-year-olds received oral health examination (174).

The General Protocol for Child Protection from Abuse and Neglect was adopted for implementation by the Government of the Republic of Serbia in 2005 and was developed in collaboration with the WHO and International Society for Prevention of Child Abuse and Neglect (175, 176). The document notes that neglect includes neglecting health care needs of children, such as not providing necessary dental care. However, child dental neglect is not officially reported as an independent diagnostic entity in any legal act or document concerning child neglect or abuse in the country. To date, there has only been one case of dental neglect reported (177). Also, no pediatric dentist serves as an expert on the working group that developed the National Strategy on Abuse Prevention and Child Protection in the Republic of Serbia (178). This oversight may have created a gap in the use of specific terminology, although dental neglect is an officially reportable entity. Having a specialist in dentistry as a member in the multidisciplinary team handling child neglect will improve the attention paid to dental neglect in Serbia.

#### United Kingdom

Despite the availability of free dental care to children through the National Health Service (NHS), inequality in dental caries is a persistent problem in England. Dental caries affects children whose families are subjected to socioeconomic adversities, live in deprived areas and are ethnic minorities (179). Furthermore, apparent socioeconomic inequalities in regular dental visits were also observed among children in the UK (180). This is particularly important, as it hinders early identification of those at high disease risk for prevention and timely intervention. Difficulties such as availability of dental appointments, inability to take time off work and cost of transportation have been recognized as potential contributors to inadequate regular dental visits and have led to delayed caries detection and treatment; however, others have highlighted the possibility of child dental neglect as a reason for delayed treatment of caries (181).

The National Institute for Health and Care Excellence (NICE) recognizes unexplained oral injuries and persistent failure to seek dental care for dental problems when services are available as child dental neglect (182). The NICE guidelines enumerate considerations for child dental neglect and place the responsibility of child dental neglect on parents (183). Others have criticized this approach and argued that there should be a role for dentists to communicate with other healthcare professionals and social services to tackle child dental neglect (184). In 2022, there was an apparent improvement in the training of undergraduate and postgraduate dental students to identify child neglect, communicate with different agencies and refer suspected cases of maltreatment and neglect (185).

The government has however also recognized the role of socioeconomic and environmental factors in child dental neglect and has developed guidance aimed at preventing dental caries and making healthy choices the easier choices. This included providing parental advice on breastfeeding, healthy eating and reduction of sugar consumption, increased taxation on soft drinks, targeted supervised tooth brushing, provision of toothbrushes and toothpaste by post, and targeted community fluoride varnish and water fluoridation (186). The government also has legislation and recommendations to prevent child maltreatment and neglect, which include the Children's Act 1989 (187) and the Working Together to Safeguard Children program.

### South-East Asia region

#### India

India is home to the largest child population in the world, with almost 41% of its 1.4 billion people under 18 years of age. For children below 6 years, the prevalence of ECC is about 49.6%, ranging from 41.9% to 69% in different regions (188); the prevalence of dental trauma is about 15% (189). Dental neglect is a significant problem, and causes include parental deferment of the child's treatment needs despite being aware, or the child's fear of dental treatment preventing the treatment from being done (190). Neglect is worse amongst female children, children from joint families and children of parents with low socioeconomic status (191).

Article 21 of the constitution of India (192) guarantees the right to health as a fundamental right of all citizens. The scope of Article 21 was further widened, as the Supreme Court held that it is the responsibility of the Government to provide adequate medical aid to every person and to strive for the welfare of the public at large. India is also a signatory to the United Nations Convention on the Rights of the Child and has the Children Act, 1960 (193), which provides for the care, protection, maintenance, welfare, training, education, and rehabilitation of neglected or delinquent children. There are, however, no studies that quantify the magnitude of established cases of child dental neglect. The constitutional right of the child to health has not been reflected in the oral health of children in India. Awareness of oral diseases is a key for preventing dental neglect. A specific law on the right of every child in India to oral health may change this paradigm.

#### Indonesia

Many children in Indonesia have poor oral health, and this problem continues into teenage and adult years (194). Over 90% of Indonesian children aged 5–6 years have caries (195) and the oral health of street children is very poor (196). It is estimated that approximately 22.2% of children in Indonesia experience neglect (196) driven by variables at the individual (gender, age of the child, and child's disability status) and household level (housing condition, education level and age of the head of household, household economic status, number of minor household members, parents' working status, one or both parents dead, existence of other neglected children in the household, and living arrangements).

The Law of the Republic of Indonesia Number 23 of 2002 and Number 35 of 2014 make up the primary legal framework that governs the protection of children in Indonesia (197, 198). These laws recognize the fundamental right of children to protection from violence, abuse, neglect, and exploitation, as well as the right to education, healthcare, and social welfare. The laws also recognize the importance of providing children (anyone below the age of 18 years) with access to quality health care services, nutrition, clean water and sanitation; and ensuring that children's physical, mental, and emotional well-being is protected (197, 198). The laws also prohibit any form of violence, abuse, or neglect that may harm children's physical or mental health, and imposes penalties on perpetrators (197, 198). The law, however, requires that the government provide free health care services to children in need, particularly those from low-income families, marginalized communities, and remote areas. When the government has met its obligation, a case can be established of willful failure of a parent or guardian to seek and receive treatment necessary to ensure a level of oral health essential for adequate function and freedom from pain and infection of the child. However, there is no specific data on the neglect of children, nor is there any on child dental neglect. This highlights an area that needs further research and policy attention in the country.

### Western pacific region

#### Mainland China

Dental caries among children is a major public health problem in Mainland China. Over 70% of 5-year-olds and about 40% of 12-year-old children experienced dental caries in 2015 (199, 200). Most of the decayed teeth were not restored. The mean DMFT score was 0.83 and the mean DT score accounted for 83.4% of dental caries experience among 12-year-old children. For the 5-year-old children, the mean caries experience score was 4.24 and the mean dt score accounted for 95.9% of dental caries experience (200). Dental neglect resulting from poor awareness of oral healthcare and poor oral health-related knowledge contributes to the high level of untreated decayed teeth (77, 201).

The 2016 Healthy China 2030 blueprint and associated action plans proposed promotion of oral health, integration of oral examination into regular physical examinations and provision of topical fluoride, pit and fissure sealing and other oral health-care measures for children (202). Also, China enacted the Law on Protection of Minors (203), which requires that stakeholders, including parents/legal guardians, schoolteachers, and healthcare workers, be responsible for the protection of the physical and mental health of minors, safeguard their lawful rights and interests, and promote their all-around development. This law implies that poor uptake of accessible oral healthcare services by caretakers of children in need of oral health care is illegal. There is, however, no country level legal instrument that directly addresses child dental neglect in China.

The establishment of cases of child dental neglect may be challenging in the short run. China has a large population of children with dental caries experience, many of whom do not routinely visit the dental clinic: about 40% of 12-year-old school children have never visited a dentist (204). This makes it difficult to establish a case of dental neglect for most affected children. When a child has no dental home, dental professionals have poor knowledge and awareness of dental neglect (201), and the general public's prioritization of oral health needs is low (199). These individual, household, and structural barriers may limit the possibility of establishing a case of dental neglect against caregivers.

#### Hong Kong

The prevalence of dental caries in preschool children in Hong Kong has remained persistently high at 55% for many years (205). Unfortunately, dental services have not kept up with this burden (206), and a staggering 95% of caries in primary dentition remains untreated (205). In contrast, the caries prevalence in 12-year-old children is lower at 23% with a DMFT index of 0.4 (207). The success of the free dental care services provided by the School Dental Care Service is believed to contribute to this lower caries prevalence in older children while low socioeconomic status has been linked to a higher prevalence of dental caries in young children (208).

The Hong Kong government adopted the United Nations Convention on the Rights of the Child in 1997, with a commitment to providing children with the highest attainable standard of health (209). Policies related to child protection and child abuse are formulated by the Child Protection Policy Unit (210), and services for children in need of protection from maltreatment are provided by the Family and Child Protective Services (211). The law holds individuals over the age of 16 years responsible for willfully neglecting a child, with potential prison sentences of ten years on indictment or three years on summary conviction (212).

Despite these regulations and efforts, the number of new child abuse cases in Hong Kong has alarmingly increased from 940 in 2020 to 1367 in 2021, with child neglect accounting for about 20% of all reported cases of abuse/maltreatment (211). Dental neglect, which can be prosecuted as child neglect, often goes unnoticed or unreported due to poor awareness and knowledge about the signs of dental neglect. It is particularly prevalent among those with low socioeconomic status (213).

## Summary of findings

As mentioned in the Methods section, we theorized that countries with developed health and social care systems [including for child dental health and child (dental) neglect] had well-defined policies to address child dental neglect. Table 1 provides a summary of the prevalence of caries, and alongside, summarizes the legal status on child dental neglect in the 26 countries and the one special administrative region studied. The side-by-side presentation of this data allows for an at-a-glance review of the data relevant to our theory. Twenty-five of the 26 countries and administrative regions have legal instruments to address child neglect. However, only two (8.0%) of these 25 countries and administrative regions had specific legal instruments on child dental neglect.

**Table 1:** Prevalence of caries and legal status on child (dental) neglect in 26 countries and a special administrative unit of China.

Country	^a^Prevalence of untreated caries in primary teeth (at age 1–9 years)	^a^Prevalence of untreated caries in permanent teeth (at age 5+ years)	Legal instrument on child neglect	Specific legal instrument on child dental neglect	Summary of content of legal instrument on child dental neglect
WHO Africa Region					
Nigeria	35.5%	23.9%	Y	N	• Not applicable
South Africa	41.0%	27.9%	Y	N	
Sudan	42.1%	35.5%	Y	N	
Tanzania	22.5%	32.7%	Y	N	
Zimbabwe	44.7%	27.0%	Y	N	
WHO The Americas Region					
Argentina	41.0%	36.9%	Y	N	• Not applicable
Brazil	46.4%	25.9%	Y	N	• Not applicable
Canada	38.9%	25.1%	Y	N	• Not applicable
Chile	46.6%	49.5%	Y	N	• Not applicable
Mexico	44.0%	23.6%	Y	N	• Not applicable
Peru	44.6%	38.2%	Y	N	• Not applicable
United States of America	42.6%	24.3%	Y	Y	• Of the 51 USA jurisdictions with Child Neglect Laws in place, eight specify “dental” in their definition of child neglect. These are: (i) New Jersey: N.J. Rev. Stat. § 3A:51–7.1; (2) New York: 18 NYCRR 433.2; (3) Oklahoma: Okla. Stat. tit. 10A, § 1–2-105; (4) Oregon: OAR 407–045-0887; (5) Pennsylvania: 23 Pa.C.S. § 6301–6385; (6) Utah: Utah Code § 78A-6–105; (7) Virginia: 22VAC40–705-30; Va. Code Ann. § 63.2–100; (8) Wisconsin: Wis. Adm. Code DHS § 88.02 • Forty-five US jurisdictions (88%) include medical neglect in their definitions of child neglect, with several jurisdictions considering dental neglect as a part of medical neglect. • All 51 US jurisdictions protect mandated reporters (i.e., dentists) from civil and criminal liability when a report is made in “good faith”. • All 51 US jurisdictions have sanctions for a mandated reporter's failure to report neglect. These include the possibility for parents to face criminal charges which can result in fines, probation, or even imprisonment; child protective services may become involved, and the child may be removed from the parent's custody if it's determined that the neglect poses a serious risk to the child's health and well-being; Courts may order the parent to provide necessary dental care for the child; parents may be required to attend parenting classes to learn about proper child care, including dental hygiene. • The specific actions vary by jurisdiction. However, the primary concern in such cases is the well-being and health of the child, and the legal system aims to ensure that neglected children receive the necessary care and protection. • A conviction for child dental neglect can have long-term consequences, affecting the parent's record and future legal matters. • Cruelty to a child attracts an offence range from community order to 8 years’ custody, with a maximum of 10 years’ custody'. • Sanctions vary by jurisdiction and include fines, imprisonment, or both (214).
Uruguay	42.9%	37.6%	Y	N	• Not applicable
WHO Eastern Mediterranean Region					
Egypt	44.1%	30.4%	Y	N	• Not applicable
Iran	46.7%	33.6%	Y	N	
Libya	44.1%	36.0%	N	N	
Jordan	42.8%	38.5%	Y	N	
Qatar	46.0%	33.6%	Y	N	
Saudi Arabia	53.2%	38.8%	Y	Y	
WHO Europe Region					
Italy	36.1%	29.6%	Y	N	• Not applicable
Latvia	47.5%	35.7%	Y	N	
Serbia	49.1%	41.1%	Y	N	
United Kingdom	19.5%	30.6%	Y	Y	• The legislation to prevent child maltreatment and neglect includes the Children Act 1989 (215); and the recommendations are in the Working Together to Safeguard Children. • The National Institute for Health and Care Excellence (NICE) highlights two areas of dental neglect: unexplained oral injuries in the child and persistent failure to seek dental care for children's dental problems. • Specifically, the 2017 NICE guidelines requires that neglect should be considered if parents or carers have access to but persistently fail to obtain treatment for their child's dental caries (tooth decay). • If the general practitioners had concerns have been raised about the wellbeing or safety of a child social services will get involved taking care of the child in the parent's home while providing support for the family to improve the child's circumstances. • Only where this is not possible, and the child is deemed to be at risk of significant harm should social services take steps to take the child from the parents. • The NICE guidelines place the responsibility of child dental neglect on parents.
WHO South-East Asia Region					
India	43.3%	28.8%	Y	N	• Not applicable
Indonesia	46.9%	28.8%	Y	N	
WHO Western Pacific Region					
China	47.2%	24.6%	Y	N	• Not applicable
Hong Kong	No data	No data	Y	N	

^a^ World Health Organization (WHO) Global oral health status report 2022: towards universal health coverage for oral health by 2030. Available at: https://www.who.int/publications/i/item/9789240061484. Accessed: 10 April 2023.

The data does not reveal any discernible correlation between the prevalence of untreated ECC and the presence of specific legal measures addressing child dental neglect. Nevertheless, in the two countries where legal provisions for child dental neglect exist, these measures outline the responsibilities of general practitioners in identifying instances of dental neglect and involve social protective agents to enhance the well-being of affected children. In both countries, the social workers are involved in managing the child before prosecution, and sanctions for child dental neglect can be as severe as been held in custody. The involvement of social protective agents consistently aims to address the needs of the affected child.

## Discussion

This policy analysis indicates that while many countries may have laws pertaining to child neglect, specific laws addressing the failure of caregivers to seek and provide adequate oral care and treatment for children as child neglect are uncommon. However, child neglect laws can be interpreted to encompass cases of child dental neglect, provided other corroborating conditions are met. In countries where the government has not adequately addressed barriers that limit children's access to oral healthcare, establishing a case of child dental neglect may prove challenging. These barriers include the unavailability of dental care, limited affordability of dental services, dentists' lack of awareness and competency to identify, manage, and report cases of child dental neglect, and a general lack of public awareness regarding the significance of oral health, the dental care system for children, and the legal implications of failing to provide oral healthcare. In high-income countries where legal instruments exist, the supporting environment does not appear to offer additional protection against the risk of untreated dental caries in children.

We hypothesize that having specific laws on child dental neglect may not provide significant protection for children against the risk of untreated caries. However, in countries where such laws exist, it is more likely that the government will establish supportive systems and structures to enhance children's access to dental care. These legal mandates for oral health care may encourage regular dental visits for checkups and diagnostic tests, thereby reducing the risk of caries at the individual level. Moreover, the existence of specific laws on child dental neglect may lead to public education on the legal responsibilities of parents and caregivers to ensure children's access to oral health care, the training of dentists in diagnosing and reporting cases of neglect, and the training of the judiciary to handle cases related to child dental neglect. This comprehensive approach can be referred to as a “social ecosystem” that promotes preventive dental care for children. Furthermore, in situations where specific laws on dental neglect are in place, but the government is not fulfilling its responsibilities, advocates can actively campaign for the establishment of systems and structures to protect the oral health of children and minimize neglect. This proactive stance can lead to improved access to dental care for children and better oral health outcomes.

Creating social ecosystems to prevent child dental neglect fits well within a human rights framework. The right to health is a fundamental human right protected and guaranteed by laws, as identified by this study. Oral health is an integral component of general health; without it, there can be no sound state of overall health. However, whether the right to health includes the right to oral health remains unresolved (216), we the authors believe that the right to health includes oral health. Where the right to oral health for children is guaranteed by legislation, the evidence highlighted in Table 1 seems to suggest that this is not enough. A social ecosystem for the prevention of child dental neglect, inclusive of the law, is needed. However, parents need to be educated about their responsibility to seek dental care for their children, particularly where free dental services are available. The competency of dental practitioners to identify, report and manage child dental neglect needs to be built. Public health authorities need to support and maintain an environment that promotes health and develop preventive dental programs for those who are overwhelmed by adverse socioeconomic conditions. Laws ensure that rights are protected and laws against dental neglect ensure that children's rights to oral health care is considered important enough for systems to be established and processes to be instituted.

We acknowledge that the right to oral health is a complex concept that has normative, philosophical, and practical dimensions, and its implementation is dictated by available resources and local priorities based on contextual factors (217). Also, the neurological, biological, and behavioral sequelae of neglect are driven by the chronicity, severity, and timing of neglect experiences (4). This complexity suggests that the determination of child neglect should be on a case-by-case basis, taking into consideration all the nuances that influence the establishments of such a case. These nuances may create a gap between the existence of laws and policies that protect children from dental neglect, and their implementation. For example, where dentists are mandated to establish the case of dental neglect, care reporting will be very low when the number of children who utilize oral health facilities is low. Children experiencing dental neglect will be less likely to be detected in the dental clinic, as they will likely only visit when there is an extreme need for dental care services. Also, in countries where the judiciary system is weak and corrupt, police reporting of child dental neglect will be low, and prosecuting cases may be compromised. Yet, deliberate actions need to be taken to safeguard the health, wellbeing, and welfare of every child by maintaining their oral health. These country specific nuances may imply that the operationalization of child dental neglect may differ from country to country.

The influence of culture on child management adds another layer of complexity to diagnosing child dental neglect and impacts how communities perceive such cases. Parenting behaviors are culturally shaped, and definitions of neglect vary across different cultures and societies (218). In some societies where poverty and limited access to dental care prevail, the lack of provisions for oral health care affects the entire community, making what may be otherwise defined as child dental neglect more of a community-level issue rather than solely the responsibility of neglectful parents as would be the case in a well-provisioned community (218). These cultural and societal nuances can impact how dental neglect is perceived, potentially leading to it being seen as a societal problem with health impact rather than a criminal act. Unfortunately, there is no consensus in the literature on the contextual definition of child dental neglect, making it challenging to analyze the factors that may influence the criminalization of such cases.

In addition, although child dental neglect may exist in isolation, untreated oral diseases can be a sign of broader child neglect and exposure to adverse experiences (77). Family isolation, lack of financial support, transportation difficulties, parental ignorance, parental psychopathology, poverty, or a lack of perceived value of oral health may all contribute to a failure to seek or obtain proper dental care (109, 119). Child dental neglect may be an indicator of child maltreatment (220), with children who are maltreated likely to have 2–8 more teeth with caries experience than children who are not maltreated (221, 222).

Ironically, although laws should protect the interests of children, their use may jeopardize public health efforts to meet children's oral health needs. Laws that criminalize child dental neglect assign blame and may not be strong enough to prevent harm (223), as the parent may be living in a social context that is disempowering, or they lack the tools to act on behalf of the child's oral health wellness and wellbeing (224). Children then experience deleterious chronic stress and adverse childhood events that further reduce their quality of life in addition to the impact on oral diseases they are suffering from (224). This collateral damage may result from the criminalization of dental neglect in countries where the experience of adverse childhood events is already high, as criminalization may have a more negative than positive impact on the child's long-term wellbeing. A viable alternative is creating or strengthening public health systems to meet the child's oral healthcare needs. Children who experience dental neglect most likely come from disadvantaged and vulnerable communities (225), which may do better with access to support rather than criminalization.

The intricate interplay of various factors complicates the establishment of a criminal case for child dental neglect, making it challenging to assess the effectiveness of legal measures in curbing untreated dental caries, a primary objective of this study. To begin with, there seems to be very few countries with explicit legal provisions addressing child dental neglect, thereby restricting the collection and analysis of pertinent data concerning child dental neglect. In cases where legal instruments do exist, the availability of analyzable data is exceedingly scarce, posing considerable difficulties in evaluating the effectiveness of such laws on a broader scale. While it's conceivable that these laws might be effective at the household level, there is minimal accessible literature providing evidence in this regard. Consequently, there is a substantial need for further research to address these gaps resulting from limited evidence, which is essential for advocating the implementation of specific laws pertaining to child dental neglect.

In the interim, combining a public health approach with a human rights-based approach, inclusive of an efficient criminal justice system to deal with dental neglect, may be more effective to control children dental neglect. The public health focus brings an analytic approach to identifying risk factors that then become the focus of preventive interventions. This may be more effective for the primary prevention of crimes than the use of punitive criminal laws (226). Such public health approach includes the use of outreach dental clinics to provide oral health services for disadvantaged and vulnerable communities (227). Unlike the criminal justice system that is reactive and punitive, the public health response is proactive and preventive. Also, the criminal justice system in many countries is ineffective in criminal behavior control, as the system is tainted from the point of arrest to investigation, arraignment in court, case hearing, verdict, and execution of court verdict (228).

In summary, the neglect of a child's oral health when caregivers or parents fail to seek and provide appropriate oral care and treatment is a matter that requires attention. However, addressing child dental neglect and considering whether it should be a criminal offense is a multifaceted issue with normative, philosophical, and practical aspects. It necessitates a careful examination of country-specific circumstances to establish the conditions under which a criminal case can be established. Both public health initiatives and the criminal justice system should, at the very least, prioritize the prevention of untreated dental caries due to its severe consequences, which include stunted physical growth and development, impaired psychological and cognitive development, adverse neurodevelopmental outcomes, and diminished quality of life (229, 230). When public health systems are sufficiently equipped to facilitate children's access to oral healthcare, then criminal penalties may be imposed on caregivers who neglect to seek oral health treatment, whether for preventive or curative purposes, for children with dental caries or other oral health needs. The extent of punitive measures should be tailored to the specific context.

This study has several limitations that should be acknowledged. Firstly, this was a narrative literature review, which may not offer a fully exhaustive and detailed understanding of the issues concerning child dental neglect and oral health problems (231). However, the review effectively fulfills its objective of providing an overview of a topic that has not been extensively explored before. The overview was, however, limited to 26 (12.7%) of the 204 countries in the world, and these countries were conveniently selected, with the number of countries not being proportionately distributed across regions. Despite these limitations in the sampling procedure, the information provided by the study sample still offers an insightful overview of a social and public health issue that may be interplaying to exacerbate societal problems.

## Conclusion

This policy review indicates that different countries have varying legislative approaches to addressing child dental neglect, and that legislative efforts alone may not be sufficient to solve this problem. A more comprehensive approach that uses a mix of public health and human rights approaches may be more successful. However, it seems that laws and legislation that prohibit child dental neglect increase the prospect of building a social ecosystem that may reduce children's risk to caries at the individual level. Where there are laws that prohibit child neglect without explicit focus on child dental neglect, it is unlikely that there will be a social ecosystem that prevents child dental neglect. Also, the social context may inform how child dental neglect is defined and operationalized. Therefore, we recommend that countries set their own nuanced definition of child dental neglect and develop a social ecosystem and legal instruments specific to country context without undermining the right of the child to oral health.
